# Predicting Parkinson's Disease Genes Based on Node2vec and Autoencoder

**DOI:** 10.3389/fgene.2019.00226

**Published:** 2019-04-02

**Authors:** Jiajie Peng, Jiaojiao Guan, Xuequn Shang

**Affiliations:** School of Computer Science, Northwestern Polytechnical University, Xi'an, China

**Keywords:** PPI network, Parkinson's disease, deep learning, node2vec, feature representation

## Abstract

Identifying genes associated with Parkinson's disease plays an extremely important role in the diagnosis and treatment of Parkinson's disease. In recent years, based on the guilt-by-association hypothesis, many methods have been proposed to predict disease-related genes, but few of these methods are designed or used for Parkinson's disease gene prediction. In this paper, we propose a novel prediction method for Parkinson's disease gene prediction, named N2A-SVM. N2A-SVM includes three parts: extracting features of genes based on network, reducing the dimension using deep neural network, and predicting Parkinson's disease genes using a machine learning method. The evaluation test shows that N2A-SVM performs better than existing methods. Furthermore, we evaluate the significance of each step in the N2A-SVM algorithm and the influence of the hyper-parameters on the result. In addition, we train N2A-SVM on the recent dataset and used it to predict Parkinson's disease genes. The predicted top-rank genes can be verified based on literature study.

## 1. Introduction

Parkinson's disease (PD) is a neurodegenerative disease, which is common in the elderly population and has an average age of onset of 60 years. The exact causes of this pathological change are still unclear. Genetic factors, environmental factors, aging, and oxidative stress may be involved in the degenerative death of PD dopaminergic neurons (Urbach-Ross and Thiruchelvam, 2010). Studies have shown that the occurrence of human diseases is rarely caused by a single gene, and most diseases are related to multiple genes (Barabási et al., 2011). Currently, there are 178 genes known to be associated with Parkinson's disease based on the NCBI (National Center for Biotechnology Information) website. Lots of genes related to Parkinson's disease still have not been discovered. The identification of genes associated with Parkinson's disease will enhance our understanding for Parkinson's disease, help us uncover the underlying molecular mechanisms of disease and aid us to diagnose disease. Therefore, it is valuable to develop a method that can predict genes associated with Parkinson's disease.

In recent years, many methods have been proposed to predict genes associated with diseases (Peng et al., 2017a,b; Cheng et al., 2018a; Hu et al., 2018; Liao et al., 2018). As more and more biological data can be utilized, it is possible to identify candidate genes based on computational methods. Comparing with *in vivo* or biochemical experimental methods, which can be extremely costly and time-consuming, computational approaches are more efficient and can guide the *in vivo* experiment. Most of existing computational methods are based on the guilt-by-association hypothesis (Cheng et al., 2018b; Peng et al., 2018b). The assumption is that genes associated with the same or similar diseases tend to accumulate in the same neighborhood of the molecular network. Therefore, a key step is to measure the distance between candidate genes and known disease genes in the protein-protein interaction (PPI) network. Lots of methods have been developed recently (Sharan et al., 2007; Wang and Marcotte, 2010).

One simple way is to determine if the two proteins are directly connected in the PPI network, so called direct neighbor counting. Oti et al. (2006) predicted genes associated with diseases by counting the number of known causative genes in their direct network neighbor. However, since two proteins that do not directly connect in PPI network are also likely to be involved in the same biological pathway, some researchers use the shortest path-based method to evaluate the association of two proteins. Krauthammer et al. (Michael et al., 2004) use this method to predict genes associated with Alzheimer's disease, and the results indicate that the genes predicted by this method are consistent with manually curated candidates. However, both methods can only capture local information of the network. In order to extract global information of the genes in the PPI network, some global methods have been proposed, such as Random Walk with Restart (RWR) (Peng et al., 2018c) and the diffusion kernel. Li et al. (Yongjin and Patra, 2010) show that these global information-based methods perform better than local information-based measurement.

In this paper, we propose a new algorithm called N2A-SVM (Node2vec Autoencoder-Support Vector Machine) to predict genes associated with Parkinson's disease. The contributions of our work are as follows:

- N2A-SVM is able to capture global topology information of a gene based on Node2vec method.
- N2A-SVM learns low-dimensional representation for each gene using a deep neural network model.
- N2A-SVM performs significantly better than existing methods.

## 2. Method

N2A-SVM consists of three steps. In the first step, node2vec is used to extract the vector representation of each gene in the PPI network. In the second step, autoencoder is used to reduce dimension of the obtained vector. Finally, we use a machine learning method, named SVM, to predict the genes associated with Parkinson's disease. The detail in the key steps of the N2A-SVM is shown in the rest of section.

### Step1. Extracting Feature Representation of Genes

Node2vec is a flexible neighborhood sampling strategy which allows us to smoothly interpolate between BFS (Breadth First Search) and DFS (Depth First Search). This method is implemented by developing a flexible biased random walk procedure that can explore neighborhoods in both BFS and DFS fashion (Grover and Leskovec, 2016). Node2vec defines a random walk with two parameters *p* and *q*. Let the current random walk position be node *v*. Let the position at previous step be node *t*. In order to determine the next position, the transition probabilities π_*vx*_ on edges (*v*, *x*) leading from *v* should be evaluated. We set the unnormalized transition probability as π_*vx*_ = α_*pq*_(*t, x*)·*w*_*vx*_. Specifically, α_*pq*_ is defined as follows.

(1) $$\;\alpha_{pq}=\{\begin{array}{cccc} \frac{1}{p} & d_{tx} & = & 0\\ 1 & d_{tx} & = & 1\\ \frac{1}{q} & d_{tx} & = & 2 \end{array}$$

where *d*_*tx*_ defines the shortest distance between node *t* and node *x*, and the value of *d*_*tx*_ must be 0, 1, or 2.

**Figure 1:**
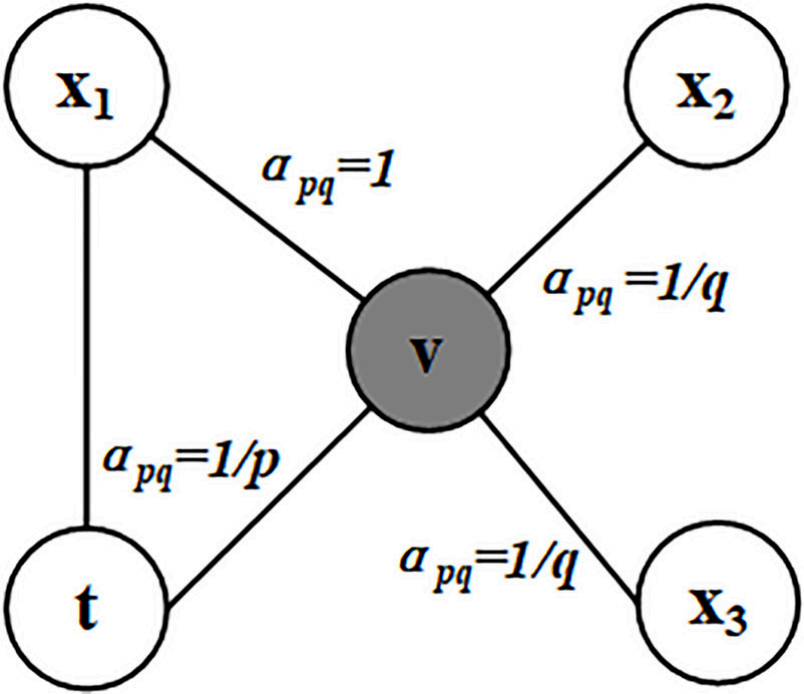
Illustration of node selection in node2vec algorithm. In this figure, the current position of random walk is at the node v and the previous step is at the node *t*. The neighbors of *v* are *x1, x2*, and *x3*. The values of *a*_*pq*_ are calculated based on the distances between the neighbors of *v* and *t*.

The parameter *p* controls the possibility of revisiting a node during the random walk. When the value of *p* is high, the nodes that have been visited will rarely be sampled. This strategy encourages moderate exploration and avoids 2-hop redundancy in sampling. On the other hand, if the value of *p* is low, it would lead the walk to backtrack a step (Figure 1) and this would keep the walk “local” close to the starting node *u*.

Parameter *q* allows the search to differentiate between “local” and “global” nodes. As shown in Figure 1, if *q* > 1, the random walk has a greater probability of sampling the nodes around the node *v*. Such walks can get a local view of the underlying graph. BFS samples nodes within a small locality. In contrast, if *q* < 1, the random walk will go farther away from *v*, which can get more global features information. Therefore, the distance between the sampling node and the given source node *u* is not strictly increased. But in turn, the measurement benefits from the superior sampling efficiency of preprocessing and random walk. In this article, we get a 512-dimensional vector representation of each gene in the PPI network via the node2vec algorithm.

### Step2. Learning the Low Dimension Representation of Features

Currently, commonly used linear dimensionality reduction methods are Principal Component Analysis (PCA), Independent Component Analysis (ICA), and Factor Analysis (FA). These dimensionality reduction methods perform well when high-dimensional datasets have linear structure and Gaussian distribution. However, when datasets are highly distorted in high-dimensional space, these methods are difficult to find nonlinear structures embedded in datasets and restore the inner structure. Therefore, we use autoencoder (Peng et al., 2018a) for low dimension feature learning in this step.

**Figure 2:**
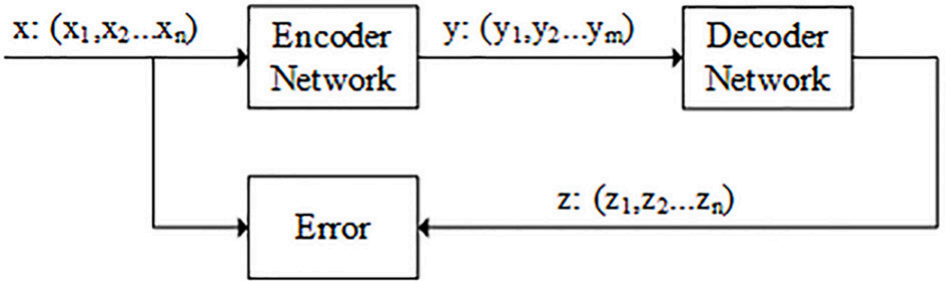
Flowchart of autoencoder. The data x is the n-dimensional feature vector that obtained in the previous step. y is the output of the encoder network. Usually, the dimension of y is smaller than dimension of x (m < n). z is the output of the decoder network and its dimension is the same as x. The model is optimized by minimizing the difference between x and z.

The autoencoder is composed of two components: encoder and decoder. The encoder belongs to the dimension reduction part, which is used to dimensional reduction. The decoder network belongs to the reconstruction part, which is the inverse of the encoder network and restores low-dimensional representation to original input data. There is also a code layer between encoder and decoder. The code layer is the key part of autoencoder network (see Figure 2).

The workflow of the autoencoder includes several steps: firstly, the weights of encoder and decoder network are initialized; secondly, the autoencoder network is trained by minimizing the error between the input and output (Hinton and Salakhutdinov, 2006). N2A-SVM algorithm uses autoencoder for data de-noising and data dimensionality reduction. Tensorflow is used to implement autoencoder.

### Step3. Predicting Parkinson's Disease Genes

The Parkinson's disease gene prediction can be considered as a classification task with two labels. We use Support Vector Machine (SVM) (Schuldt et al., 2004) algorithm to solve this bi-classification problem. For classification, SVM constructs a hyperplane or set of hyperplanes in a high-dimensional space to classify genes with different labels.

Genes associated with Parkinson's disease are considered as positive set. We randomly select genes not associated with Parkinson's disease as negative set. The negative set has the same size as positive set. We used ten-fold cross validation in the evaluation test.

## 3. Results and Discussion

In this section, we evaluate the performance of four methods of N2A-SVM, RWR (Yongjin and Patra, 2010), Shortest Path Length (SPL) (Michael et al., 2004), and Euclidean distance (ED) (Díaz-Uriarte and Alvarez de Andrés, 2006) on predicting genes associated with Parkinson's disease. RWR is a method that are widely used in network-based disease gene prediction. The ED and SPL method are used in path-based disease gene prediction. We also test the effect of each step and different parameters of the N2A-SVN algorithm on the performance of the algorithm. Finally, we apply N2A-SVM to predict new Parkinson's disease genes. The result shows that some of the genes predicted by the N2A-SVM algorithm are supported by existing literature.

### Performance Evaluation on Parkinson's Disease Gene Prediction

**Figure 3:**
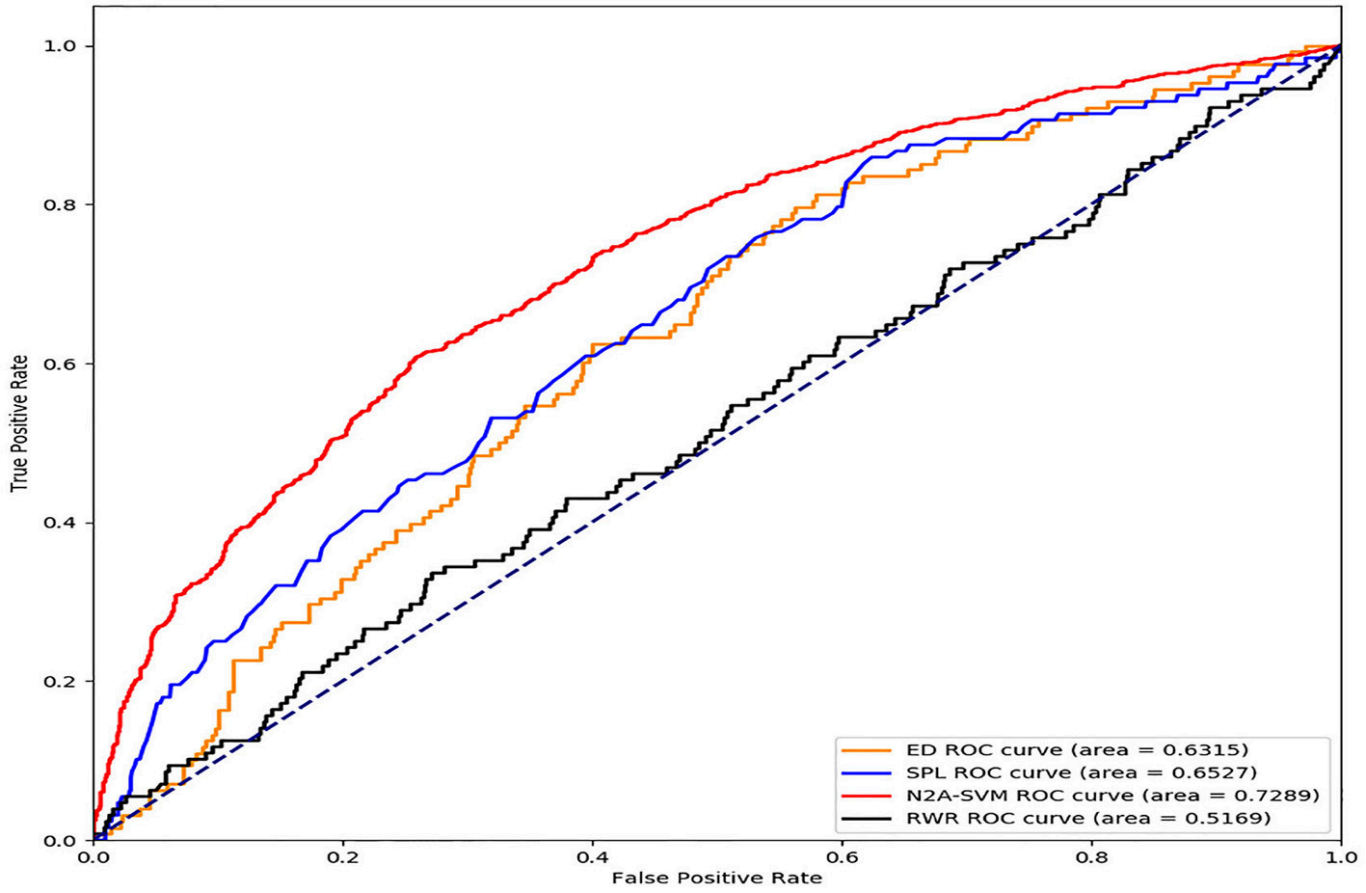
Performance comparison of the four methods (ED, SPL, RWR, and N2A-SVM).

We download genes related to Parkinson's disease from the ClinVar (https://www.ncbi.nlm.nih.gov/clinvar/). After removing deduplication, we get 178 genes associated with Parkinson's disease. In addition, we use the PPI network that is also used in (Menche et al., 2015). The network contains 13,460 nodes and 141,296 edges. In the Euclidean distance-based approach, we calculate the mean of the distances between each gene not associated with Parkinson's disease in the PPI network and all known genes associated with Parkinson's disease. Moreover, the SPL method achieves the calculation of the shortest path length between the Parkinson's disease related gene and genes that do not relate to Parkinson's disease. In addition, in the RWR-based method, we obtain the diffusion state of each gene based on the probability matrix. The AUROC (Area Under the Receiver Operating Characteristic curve) scores of the tested methods are shown in Figure 3. The result shows that the AUROC score of N2A-SVM (0.7289) is the highest, while the score of the second best method is 0.6527.

**Figure 4:**
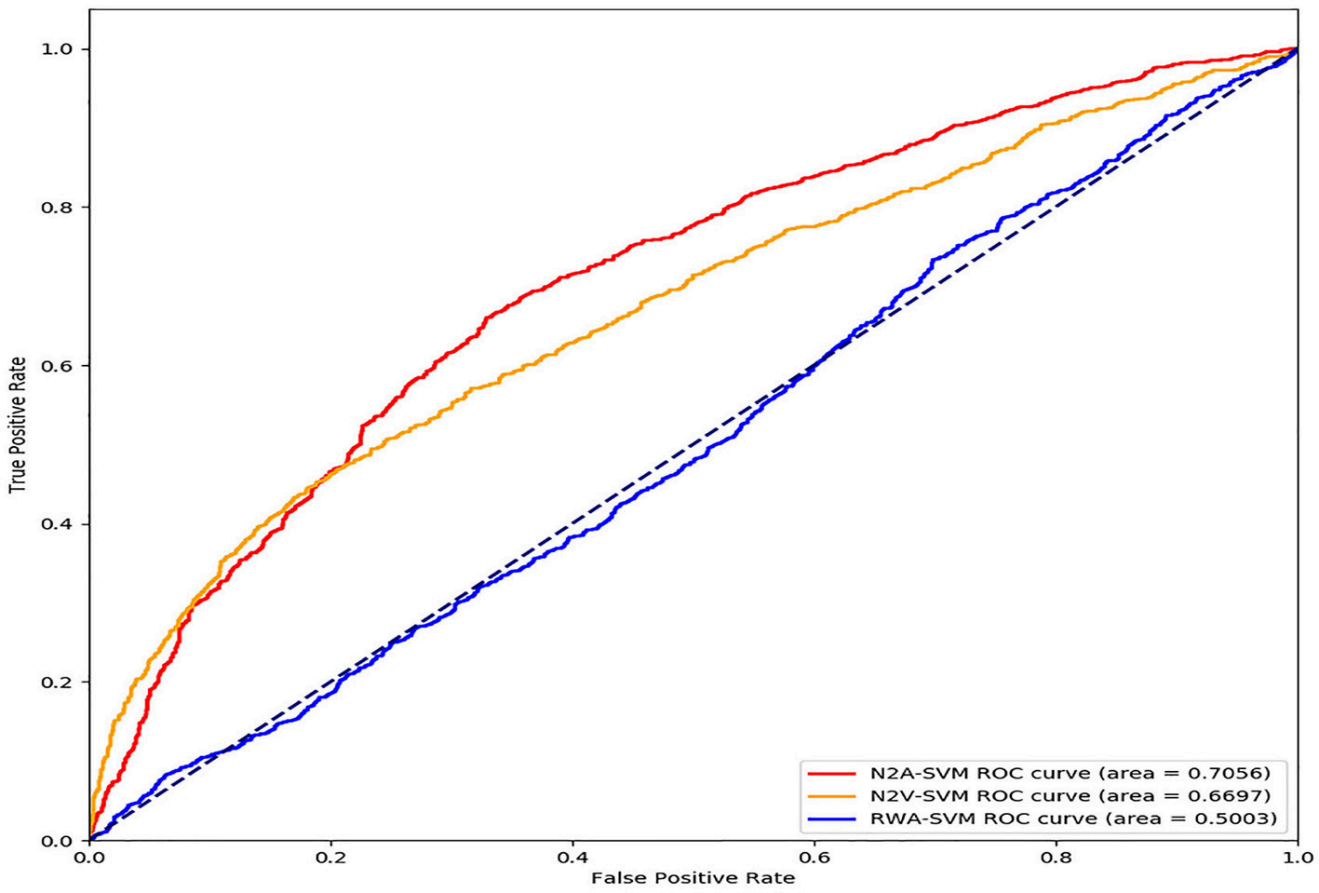
Performance evaluation by modifying each step of N2A-SVM algorithm.

In order to test the impact of each step of the N2A-SVM algorithm on the performance, we test two variations of N2A-SVM. In the RWA-SVM, we first use the RWR algorithm to obtain the features representation of each gene. The number of feature dimensions for each gene is 13,460, which is the same of the number of genes involved in the PPI network. Then, the autoencoder is used for dimensionality reduction. Finally, SVM is used for Parkinson's disease gene prediction. In the N2V-SVM, we verify the effect of the step of dimension reduction on the prediction results. We directly use the node2vec method for feature extraction, and the obtained 512-dimensional feature vector is used as the input of the SVM classification algorithm. By comparison of three methods, the result shows that N2A-SVM performs better than RWA-SVM and N2V-SVM. It is indicated that each step in the N2A-SVM algorithm is crucial to the final prediction (see Figure 4).

### The Impact of Different Parameters on Disease Gene Prediction

**Figure 5:**
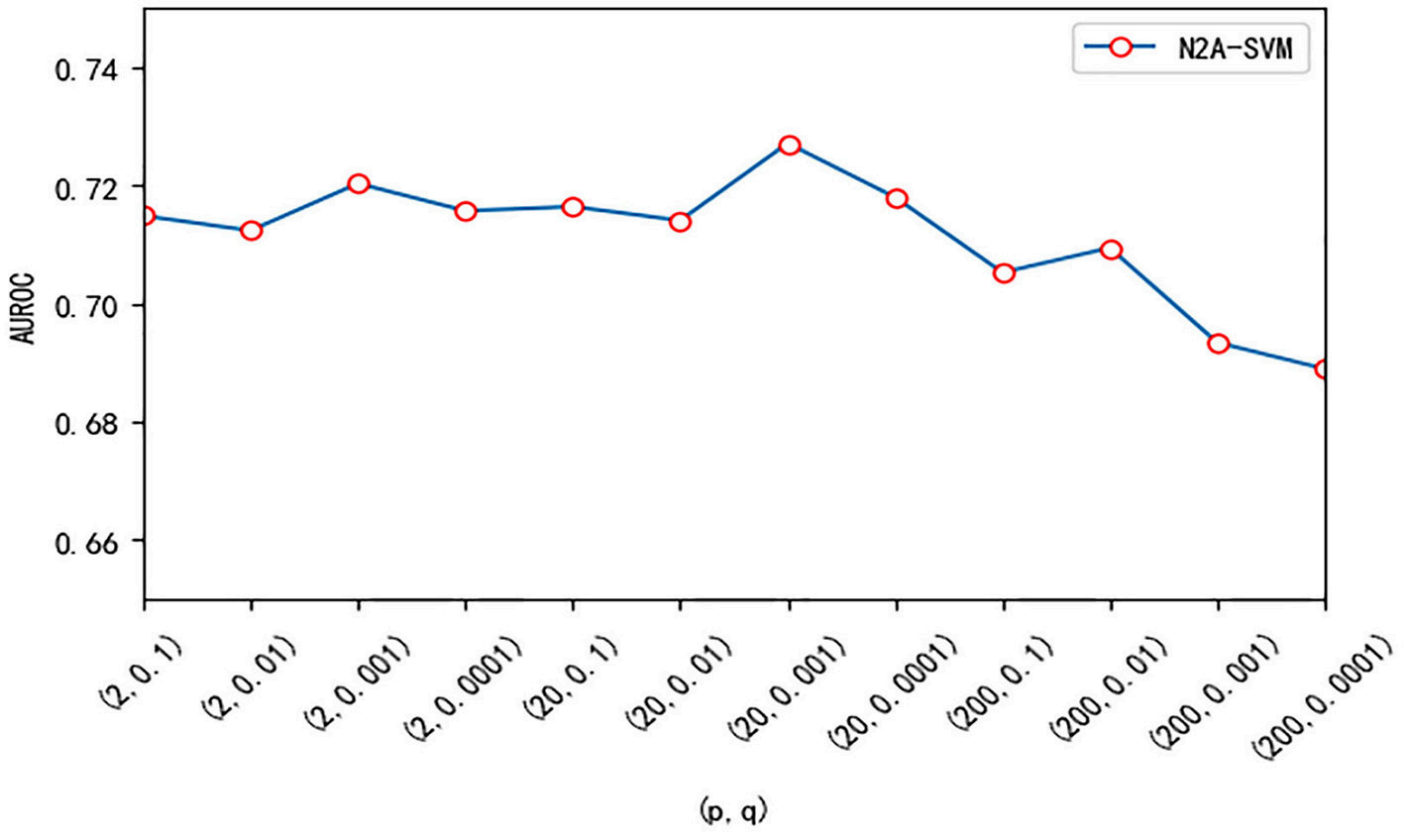
The effect of different (*p, q*) on the performance of N2A-SVM. The value of p is one of 2, 20, 200, and the value of q is one of 0.1, 0.01, 0.001, 0.0001.

**Figure 6:**
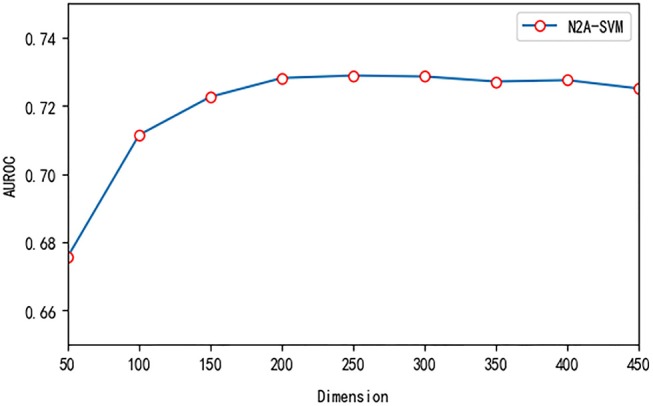
The effect of different feature dimensions on the performance of N2A-SVM.

We test three important parameters involved in our algorithm: *p*, *q* in the node2vec algorithm and the dimension size of feature obtained from autoencoder. For the parameter test, we fix one parameter and vary other parameters. *p* and *q* are related to the random walk process in the node2vec algorithm. We use different values of *p* and *q* and test their effect on the performance. Overall, the performance of the algorithm is robust to the parameter *p* and *q*. The value of AUROC varies between 0.69 and 0.73 (see Figure 5). We also test the effect of the features dimension of each gene after dimensionality reduction using the autoencoder algorithm. As the dimension increases, we find that the value of AUROC gradually becomes larger and tends to be stable when the number is larger than 200 (Figure 6).

### A Case of Predicting New Parkinson's Disease Genes

Finally, we use the N2A-SVM algorithm proposed in this article to predict the genes associated with Parkinson's disease. After training the model, we use the model to predict new Parkinson's disease genes that do not included in the database. We first rank all the genes based on the probability predicted by the trained model. We select the top ten genes and look up them in the literature. The 10 genes are TRIM63, MT-ND4, NDUFB5, NDUFA6, MYOZ1, DHDDS, PICK1, CIC, PARK2, and HGS. Based on literature review, we find that some of these genes have been reported to be associated with Parkinson's disease. PICK1, PARK2, MOYZ1 are reported in He et al. (2018) and Padmaja et al. (2012). In addition, three genes, MT-ND4, NDUFB5 and NDUFA6, affect the synthesis of mitochondrial complex 1 associated with Parkinson's disease (Rodenburg, 2016; Talebi et al., 2016). Among the remaining four genes, DHDDS is associated with the onset of epilepsy (Hamdan et al., 2017). It can be found in UniProtKB that the CIC gene is involved in the formation of the central nervous system and the development of the brain. HGS is associated with multiple sclerosis (MS) (Igci et al., 2016), an inflammatory disease of the central nervous system caused by genetic and environmental factors. From the 2019 version of the gene table of neuromuscular disorders (Bonne et al., 2018), we find that the TRIM63 gene is involved in neuromuscular diseases. In total, TRIM63, DHDDS, CIC, and HGS are all associated with neurological diseases.

## 4. Conclusions

Identifying genes associated with Parkinson's disease is of great importance for the treatment of Parkinson's disease. In this article, we present a new algorithm, named N2A-SVM, to predict Parkinson's disease gene. N2A-SVM includes three steps: (1) extracting the vector representation of each gene in the PPI network using node2vec; (2) reducing dimension of the obtained vector using autoencoder; (3) predicting the genes associated with Parkinson's disease using SVM. We compare N2A-SVM with RWR and distance-based method and prove that N2A-SVM performs better than the compared methods. In addition, we use the N2A-SVM algorithm to discover new genes associated with Parkinson's disease. Ten genes most likely to be associated with Parkinson's disease have been proved by literature study. In the future, we will use this method in the prediction of other related diseases, and hope to apply biological experiments to verify the results.

## Data Availability

All datasets generated for this study are included in the manuscript and/or the supplementary files.

## Author Contributions

JP and XS designed the algorithm. JG implemented the algorithm. JP and JG wrote this manuscript. All authors read and approved the final manuscript.

### Conflict of Interest Statement

The authors declare that the research was conducted in the absence of any commercial or financial relationships that could be construed as a potential conflict of interest.
